# Natural Phytochemicals Derived from Gymnosperms in the Prevention and Treatment of Cancers

**DOI:** 10.3390/ijms22126636

**Published:** 2021-06-21

**Authors:** Tayyebeh Ghaffari, Joo-Hyun Hong, Solmaz Asnaashari, Safar Farajnia, Abbas Delazar, Hamed Hamishehkar, Ki-Hyun Kim

**Affiliations:** 1 Drug Applied Research Center, Tabriz University of Medical Sciences, Tabriz 15731, Iran; ghaffari.t@tbzmed.ac.ir (T.G.); farajnia@gmail.com (S.F.); 2 Student Research Committee, Tabriz University of Medical Sciences, Tabriz 15731, Iran; 3 School of Pharmacy, Sungkyunkwan University, Suwon 16419, Korea; ehong@skku.edu; 4 Biotechnology Research Center, Tabriz University of Medical Sciences, Tabriz 15731, Iran; asnaasharisolmaz@gmail.com; 5 Research Center for Evidence based Medicine, Tabriz University of Medical Sciences, Tabriz 15731, Iran; delazara@tbzmed.ac.ir

**Keywords:** gymnosperm, cancer, paclitaxel, traditional medicine, natural products

## Abstract

The incidence of various types of cancer is increasing globally. To reduce the critical side effects of cancer chemotherapy, naturally derived compounds have been considered for cancer treatment. Gymnosperms are a group of plants found worldwide that have traditionally been used for therapeutic applications. Paclitaxel is a commercially available anticancer drug derived from gymnosperms. Other natural compounds with anticancer activities, such as pinostrobin and pinocembrin, are extracted from pine heartwood, and pycnogenol and enzogenol from pine bark. Gymnosperms have great potential for further study for the discovery of new anticancer compounds. This review aims to provide a rational understanding and the latest developments in potential anticancer compounds derived from gymnosperms.

## 1. Introduction

Natural plant products have been used as medicines throughout human history for different purposes. Natural compounds are complex chemical molecules found in various parts of plants with pharmacological or biological activities and are used for the treatment of cancer, inflammation, infections, and other diseases [1]. In the past 30 years, approximately 61% of bioactive natural compounds have been used for cancer treatment, and 49% of these have also been used for infectious disease treatment [2]. Cancer is the second leading cause of death globally, with more than six million deaths each year [3].

The use of natural components for cancer prevention and treatment has been widely studied. Curcumin is a well-known anticancer compound found in the rhizome of *Curcuma longa*. Curcumin can promote autophagy through the AMP-activated protein kinase signaling pathway, which is related to cell death [4,5]. Another potential candidate for anticancer agents is resveratrol, a polyphenol found in peanuts, soy, and grapes. Resveratrol induces autophagy through the ceramide/Akt/mTOR pathway [6]. 6-Shogaol, an active component of ginger, inhibits cancer cell invasion by blocking the nuclear factor-κB pathway (NFκB) [7]. In addition, flavonoids have different mechanisms for cancer treatment. Cyclin-dependent kinases (CDKs) are inhibited by flavonoids, such as quercetin, silymarin, genistein, luteolin, apigenin, and kaempferol. Different types of cancers are related to the activation of CDKs due to mutations in CDK genes. Therefore, CDK inhibitors are novel agents for cancer treatment [8,9,10]. Epigallocatechin gallate is a polyphenol found in the dried leaves of green tea that initiates cancer cell apoptosis through the activation of the p38 MAP kinase [11]. Many natural products have been commercialized for cancer treatment. One of the most effective natural anticancer agents for the treatment of various cancers is paclitaxel (Taxol^®^), which was discovered in the bark of Pacific yew, *Taxus brevifolia*. Paclitaxel is a potent inhibitor of mitosis by binding to tubulin and preventing its depolymerization during cell division [12]. 

At present, interest in the use of traditional medicine is increasing, and some important anticancer drugs have an herbal origin. Gymnosperms consist of an estimated 12 families, 83 genera, and more than 1000 living species, including those utilized in the production of commercially available drugs and dietary supplements, such as paclitaxel, pycnogenol, enzogenol, pinostrobin, and pinocembrin. Gymnosperms can be a source of effective cancer drugs. Various natural compounds derived from gymnosperms are considered as potential candidates for cancer therapy. Leelamine, α-pinene, and β-pinene have been studied for their ability to inhibit the growth of cancer cell lines [13,14,15]. Other terpenoids in gymnosperms, such as limonene, α-phellandrene, β-caryophyllene oxide, γ-terpinene, longifolene, D-germacrene, verbenol, and β-ocimene, have anticancer effects [16,17,18,19,20]. Due to the anticancer potential of gymnosperms and the relatively few studies that have been conducted in this field, we provide here a comprehensive summary of natural phytochemicals derived from gymnosperms in the prevention and treatment of cancers. 

## 2. Gymnosperms in Cancer Therapy

Gymnosperms are defined as a group of plants that produce seeds that are not enclosed within an ovary or fruit. Gymnosperms include Cycadophyta (cycads), Ginkgophyta (ginkgo), Gnetophyta, and Coniferophyta (conifers). The main group of living gymnosperms is conifers, such as pines, yews, cedars, and redwoods, which are cone-bearing trees and shrubs. More than 600 species have been reported and are dispersed worldwide [21]. Conifers are one of the oldest groups of plants that are ecologically and economically the most important plant group. The leaves of many conifers, such as pines, are needle-like, but yews, cedars, and redwoods have flat, scale-like, and triangular leaves. Cycads are the next most abundant group of gymnosperms. Cycads are palm-like woody plants that make up approximately 338 species, most of which are native to tropical climates. Gnetales is the other living group of gymnosperms, consisting of 95–100 species comprising *Gnetum*, *Ephedra*, and *Welwitschia*. The smallest genus of gymnosperms is *Ginkgo*, which comprises one extant species [22].

**Figure 1:**
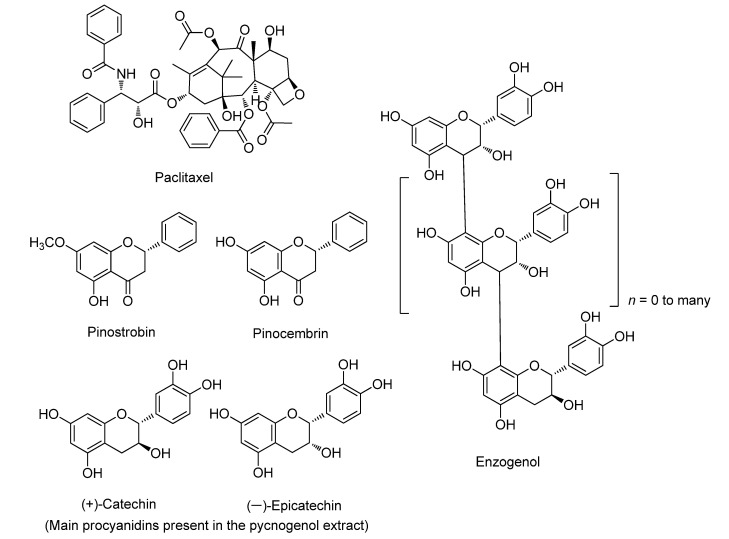
Chemical structures of the commercially available products displayed in Table 1.

Gymnosperms are reservoirs of terpenoids, phenolic components, alkanes, and alkenes. In Asia, gymnosperms are traditionally used as medicinal plants for various disease treatments. Most are used to treat stomach disorders, arthritis, fever, diabetes, cold, ulcers, and even cancer. Paclitaxel is an anticancer drug obtained from the bark of yew trees. Paclitaxel is used to treat ovarian, breast, lung, and prostate cancers. The structures of the selected commercially available compounds discussed in this review are shown in Figure 1. Leelamine from pine bark shuts off phosphoinositide 3-kinase (PI3K), mitogen-activated protein kinase (MAPK), and signal transducer and activator of transcription 3 (STAT3) pathways in cancer cells [13]. α-Pinene from pine needle oil causes a reduction in the levels of cyclin B protein in hepatoma cell lines in vitro [14]. β-Pinene is a major component of various pine essential oils and inhibits the growth of cancer cell lines [15]. Some commercially available drugs and dietary supplements [12,23,24,25,26,27,28,29,30,31,32,33] are derived from gymnosperms and their physicochemical properties (Table 1). The anticancer effects of stilbenoids in *Welwitschia* and five species of Gnetaceae were studied. The results showed that these stilbenoids had cytotoxic activity against HL60 cells [34]. Limonene prevents the assembly of microtubules in dividing cells [35]. β-caryophyllene oxide is a sesquiterpene isolated from the essential oils of various species of pine. It has an anticancer effect through the inhibition of c-Src kinase and Janus kinase 2 (JAK2) [36].

## 3. Clinically Available Natural Products

### 3.1. Paclitaxel

**Figure 2:**
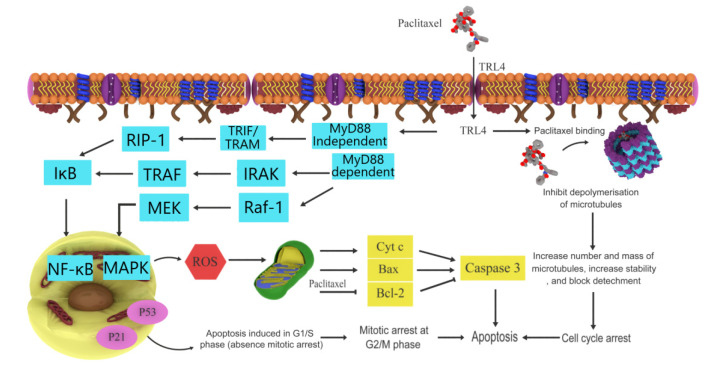
Possible mechanism of paclitaxel action. Paclitaxel targets microtubules and inhibits the depolymerization of microtubules by binding to β-tubulin and leading to cell death. Solute carrier organic anion transporter family member 1B3, which is expressed in various tumors, is the most effective influx transporter for paclitaxel. Paclitaxel induces apoptosis via reactive oxygen species production as well as p21, B-cell lymphoma-2 associated X protein, and caspase overexpression and also activates the Toll-like receptor 4/nuclear factor kappa B pathway. (adopted with modification from [12]).

Paclitaxel (Taxol^®^) is a well-known chemotherapeutic agent with a taxane structure. It is extracted from the bark of the North American Pacific yew tree, *T. brevifolia*, with wide anticancer uses, including for small-cell lung cancer, refractory ovarian cancer, Kaposi sarcoma, metastatic breast cancer, and melanoma [37]. Paclitaxel comprises a taxane ring (Figure 1) in the C4 and C5 positions and has a four-membered oxetane side ring in C13, which is an ester side chain that plays a key role in the formation of the paclitaxel-tubulin complex, blocks the progression of the cell cycle in mitosis, and stabilizes microtubules. Paclitaxel binds to the β-subunit of tubulin on the inner surface of microtubules and prevents the dissociation rate of tubulin dimers in a guanosine triphosphate-independent manner. As a result, this complex cannot be disassembled and affects the cell dynamics that are necessary for intra-cell transportation and chromosome movement during mitosis. During the metaphase of mitosis, incomplete formation of the metaphase plate leads to cell cycle arrest. Paclitaxel blocks cells in the G2/M phase of the cell cycle without disrupting the synthesis phase [38,39]. Therefore, paclitaxel is a potent cell replication inhibitor in eukaryotic cells, which ultimately causes apoptosis [40,41]. Paclitaxel induces apoptosis in prostate and breast cancer cell lines by anti-apoptotic B cell lymphoma 2 (Bcl-2) phosphorylation, consequently arresting its function [23]. Recently, paclitaxel was reported to upregulate mitogen-activated protein kinase (MAPK) in ovarian cancer [42]. Among cancer cells, p53 has a pro-apoptotic role in HCT116 colon cancer cells. However, in the presence of paclitaxel, acetylation of p53 was observed, which is more susceptible to cell death [43]. In the human breast adenocarcinoma cell line, paclitaxel induced apoptosis via higher G2/M cell-cycle arrest, reactive oxygen species (ROS) production, and mitochondrial membrane potential disruption. ROS generation and increased hydroperoxide production by nicotinamide adenine dinucleotide phosphate oxidase is considered to play a key role in the anticancer activity of paclitaxel. Upregulation of p21, Bcl-2 associated X protein (Bax), caspase-3, and caspase-9 suggests that apoptosis occurs through intrinsic pathways [23]. Sun et al. reported that paclitaxel induces apoptosis through the activation of Toll-like receptor 4 (TLR4) via the MyD88-independent or -dependent pathway and the NFκB pathway in ovarian carcinoma cells [44]. Phosphorylation of interleukin (IL)-1 receptor-associated kinases by MyD88 initiates a signaling pathway that results in the activation of MAPKs. Furthermore, TLR4, through the TIR-domain-containing adapter-inducing interferon-β adaptors, activates receptor-interacting protein, resulting in NF-κB activation. Paclitaxel treatment induces cytotoxicity, apoptosis, and growth inhibition in the cells, which was confirmed by decreased cell viability. Low doses of paclitaxel induce the enhanced expression of p53 protein without disturbing the cell cycle and its nuclear translocation with well-performed apoptotic events in breast cancer cells [45]. The mechanisms underlying the anticancer activity of paclitaxel are summarized in Figure 2.

### 3.2. Pinostrobin

Dietary flavonoids are a large group of polyphenolic molecules with low molecular weights obtained from plant sources. Several phenolic compounds have numerous health benefits, including anti-carcinogenic, antioxidant, anti-inflammatory, and antimicrobial activities. Pinostrobin, 5-hydroxy-7-methoxy flavanone (Figure 1), is a natural flavonoid that exists in *Pinus strobus, Alpinia zerumbet, Salvia texana*, and other plants [46]. Pinostrobin exhibits many therapeutic activities, such as anti-*Helicobacter pylori* and anti-herpes simplex virus-1 activity, demonstrating its antibacterial and anti-viral activity [47]. Furthermore, the ability of pinostrobin to prevent the cyclooxygenase enzyme pathway indicates anti-inflammatory potential [27]. In addition, pinostrobin has significant effects on cell cycle arrest, growth inhibition, and apoptosis in many cancers [48]. Jaudan et al. demonstrated that HeLa cells are more sensitive to pinostrobin than other cancer cell lines in vitro [27]. They also found that pinostrobin induced ROS generation and apoptosis in HeLa cells via extrinsic and intrinsic pathways. In HepG2 liver cancer cell lines, pinostrobin demonstrated moderate cytotoxicity [49]. Among the various active components isolated from *Cajanus cajan*, pinostrobin showed maximum toxicity on the T lymphoblastoid cell line derived from patients with acute leukemia, whereas in other cell lines, such as lung cancer, melanoma, and breast cancer cell lines, toxicity was lower [50]. Based on the results of these studies, pinostrobin appears to have anticancer potential.

### 3.3. Pycnogenol

**Figure 3:**
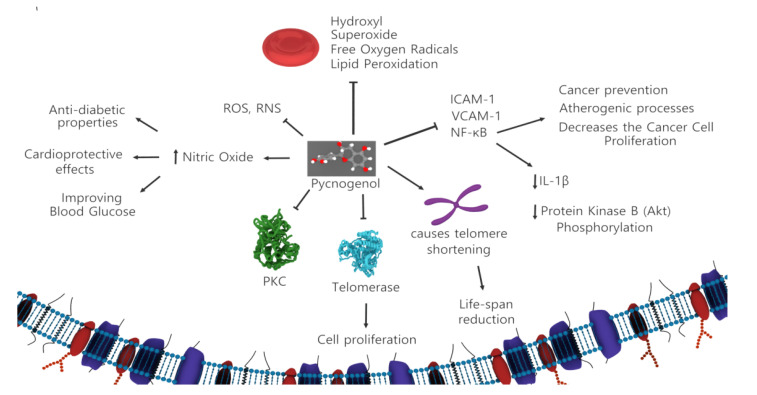
Mechanism of pycnogenol anticancer and antioxidant action. Pycnogenol can inhibit PKC and telomerase in cancer cells for life-span reduction. Furthermore, by reducing NFκB activation, VCAM-1 and ICAM-1 can prevent cancer. Pycnogenol has anti-diabetic and cardioprotective effects through increasing nitric oxide levels and reducing lipid peroxidation.

Pycnogenol^®^ is a formulation of *Pinus pinaster* (French maritime pine) bark aqueous extract with a composition of a mixture of flavonoids, mainly procyanidin compounds. Procyanidins are chain-like structures comprising catechin, epicatechin, phenolic acids, taxifolin, and cinnamic acids [51]. Pycnogenol is similar to cinnamon, grape seed extract, green tea, and cocoa bean polyphenols, which are the four most common sources of procyanidins. Procyanidins may play an important role in preventing and treating cancer [52]. Procyanidins exhibit inhibitory effects on the proliferation of certain tumor cells in vitro and in vivo. The yields of catechin from *P. pinaster* bark extract increase as the ethanol concentration of the extract increases. Catechins, major components of pycnogenol, have anti-proliferative and cytotoxic effects on pancreatic, breast, and colorectal cancer cell lines [53]. Furthermore, catechins inhibit protein kinase C and telomerase. In colon adenocarcinoma and monoblastoid leukemia cells, the presence of catechins causes telomere shortening, and chromosomal abnormalities result in life-span reduction [54]. The expression of telomerase in most tumors is a key parameter for explaining the proliferative ability of cancer cells enabled by the preservation of the tips of the chromosomes [55]. Thus, telomerase inhibition could be one of the major mechanisms underlying the anticancer effects of catechins present in pycnogenol [56]. The molecular basis of pycnogenol activity depends on the scavenging of reactive oxygen and reactive nitrogen species and it participates in the cellular antioxidant system [57,58]. Pycnogenol has cardioprotective effects by increasing blood flow through a mechanism associated with increased nitric oxide levels; it also aids in improving blood glucose control, thereby exhibiting anti-diabetic properties [59,60]. Pycnogenol can arrest hydroxyl, superoxide, and free oxygen radicals and reduce lipid peroxidation in red blood cells [61]. The role of pycnogenol in inhibiting NFκB activation, and vascular cell adhesion molecule 1 (VCAM-1) and intercellular adhesion molecule 1 (ICAM-1) expression suggests that this antioxidant compound may play a role in cancer prevention and atherogenic processes [62,63]. Abnormal activation of NF-κB has been observed in many cancers. Furthermore, the suppression of NF-κB decreases the proliferation of cancer cells. NF-κB also plays a major role in inflammatory diseases. David et al. demonstrated that pycnogenol reduced lipid peroxidation and carbonyl proteins in ascitic fluid [56]. This action may be related to pycnogenol-mediated effects that cause NF-κB inhibition, reduce IL-1β production, and decrease protein kinase B (Akt) phosphorylation (Figure 3). This study suggests that *P. pinaster* procyanidins could be a candidate for future studies on multifunctional diet-based cancer prevention approaches.

### 3.4. Enzogenol

Enzogenol^®^ is a water-soluble proanthocyanidin-rich bioflavonoid extract derived from the bark of *Pinus radiata* that contains different flavonoids and phenolic acids. Enzogenol is rich in proanthocyanidins, 80% of which include catechin, epicatechin, taxifolin, quercetin, dihydroquercetin, procyanidin dimers, trimers, oligomers, and polymers, and other phenolic acids [64,65]. In vitro and in vivo research has shown that enzogenol has high potential as an antioxidant and anti-inflammatory agent; studies have shown it to effect a decrease in the levels of cardiovascular disease markers and to protect experimental mice against tumor growth [66,67,68,69]. One study demonstrated that enzogenol is much more effective in antioxidant activity than catechin and ascorbic acid under the same conditions [70]. O’Callaghan et al. found that enzogenol elevated caspase-3 activity in a dose-dependent manner [71]. In addition, in the presence of enzogenol, the expression of the anti-apoptotic protein Bcl-2 decreased, leading to apoptosis [33]. Enzogenol has anti-inflammatory and anti-atherosclerotic effects. The anti-inflammatory effects of enzogenol are related to the downregulation of inflammatory cytokines and the inhibition of TNF-α-induced VCAM-1 and ICAM expression [68]. Enzogenol also has cholesterol-lowering properties that can prevent atherosclerosis [72].

### 3.5. Pinocembrin

**Figure 4:**
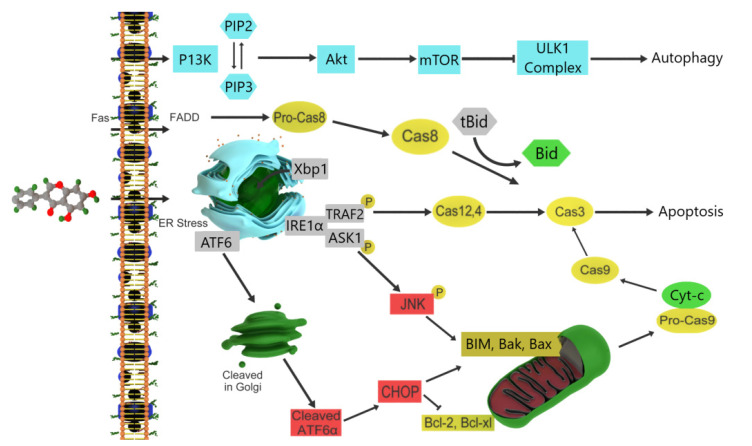
Mechanism of pinocembrin induction of apoptosis in cancer cells. In the intrinsic pathway, the expression of the pro-apoptotic proteins B-cell lymphoma-2 (Bcl-2) associated X protein, Bcl-2 homologous antagonist/killer, and Bcl-2-like protein 11 increases and that of the anti-apoptotic proteins Bcl-2 and Bcl-extra-large decreases. Cytochrome c translocates from the mitochondria to the cytosol, leading to apoptosis. In the extrinsic pathway, pinocembrin leads to apoptosis via the Fas-associated protein death domain/caspase-8/caspase-3 signaling pathway.

Pinocembrin (5,7-dihydroxyflavanone) is a flavonoid that has been isolated from several plants (Figure 1), and *Pinus pinastar* heartwood is the main source of this compound. Other sources of pinocembrin extraction are *Eucalyptus*, *Euphorbia*, *Populus*, chilca, and honey [73,74]. Pinocembrin is the most significant phytochemical among flavonoids. Its pharmacological activities include anticancer, anti-inflammatory, antioxidant, and antimicrobial properties [75]. Pinocembrin can prevent cancer or reverse disease onset by delaying or stopping the growth of cancer cells. Pinocembrin has cytotoxic effects against breast, colon, cervical, and prostate cancer cell lines [76,77]. In human colorectal adenocarcinoma (HT29) and colon cancer cell lines (HCT116), pinocembrin increases the expression of Bax protein, caspase-3 and -9, and heme oxygenase activity, as well as decreasing the superoxide anion radical and nitric oxide levels [78,79,80]. In prostate cancer cell lines (LNCaP), pinocembrin causes cell cycle arrest at the G2/M phase and is involved in the depletion of mitochondrial membrane potential before apoptosis. Subsequently, cytochrome c is released from the mitochondria and binds to apoptotic protease-activating factor 1 to activate caspase-9, which consequently activates caspase-3 [81,82]. Pinocembrin can activate the PI3K/Akt/mTOR pathway in melanoma cell lines (B16F10 and A375), which may suggest a link between pinocembrin’s pro-apoptotic effect and autophagy [83]. Phosphorylation of phosphatidylinositol-4, 5-bisphosphate by PI3K catalyzes the production of phosphatidylinositol-3, 4, 5-triphosphate (PIP3). PIP3 localized in the inner layer of the plasma membrane promotes the activation of AKT activation, followed by that of the mammalian target of rapamycin (mTOR). Subsequently, mTOR directly phosphorylates unc-51-like kinase 1 during autophagy initiation and autophagosome formation [84]. Zheng et al. demonstrated that pinocembrin can induce apoptosis in melanoma cell lines in a mitochondria-independent manner in vitro and in vivo, as well as create severe endoplasmic reticulum (ER) stress conditions in vitro. The inositol-requiring enzyme 1 (IRE1)/X-box binding protein 1 (Xbp1)/C/EBP-homologous protein (CHOP) pathway activates ER stress after pinocembrin treatment. First, IRE1 expression increases, followed by an increase in the expression of Xbp1, activating transcription factor 6, and CHOP, which are key factors in ER stress-mediated apoptosis [83]. On the other hand, IRE1 binds with TNF receptor-associated factor 2, which activates caspase-12 and -3, which leads to apoptosis. Phosphorylation of apoptosis signal-regulating kinase 1 after IRE1 activation causes c-Jun-N terminal kinase initiation of Bax, Bcl-2 homologous antagonist/killer and Bcl-2-like protein 11 activation, and B-cell lymphoma-extra-large (Bcl-xL) and Bcl-2 inhibition (Figure 4) [85]. Eventually, the Fas-associated protein death domain, as an extrinsic pathway for apoptosis, activates downstream processes, including the activation of caspases-8,-7,-6, and -3 [86]. These studies showed that pinocembrin is a strong candidate as an anticancer agent with pharmacological potential.

## 4. The Future Role of Gymnosperms in Cancer Therapy

In recent years, many studies have focused on the anticancer effects of plant derivatives. Gymnosperms have a wide variety of chemical compounds that could play a vital role in cancer treatment in the future. Some of these compounds have strong anticancer properties (Table 2). 

### 4.1. Leelamine

**Figure 5:**
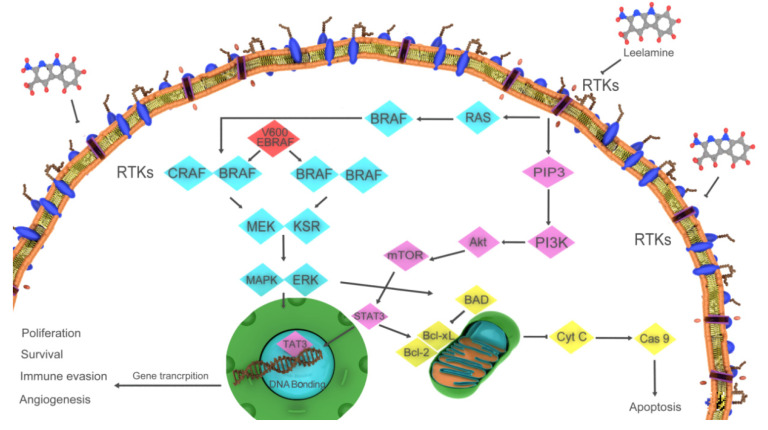
Mechanism of action of leelamine. Leelamine disrupts receptor tyrosine kinase signaling pathways, leading to a decrease in the phosphoinositide 3-kinase/protein kinase B, mitogen-activated protein kinase, and signal transducer and activator of transcription 3 signaling cascades, resulting in the reduction of B cell lymphoma (Bcl) 2 and Bcl-extra-large expression levels.

Leelamine (dehydroabietylamine) is a natural diterpene molecule found in the bark of *Pinus* species. Leelamine targets several signaling pathways involved in cancer because of the disruption of intracellular cholesterol homeostasis [91]. Cholesterol is abundant in the membrane of the mitochondria, ER, and Golgi, and plays a critical role in transport and intracellular signaling systems [92]. When leelamine accumulates in the lysosomes due to its lysosomotropic properties, translocation of cholesterol from the lysosomes to the cytoplasm is blocked. Thus, cholesterol is not available for cancer cell activities and subsequently, receptor-mediated endocytosis and autophagic flux are inhibited. This inhibition is related to receptor tyrosine kinase (RTK) signaling pathways, which can affect the PI3K/AKT, STAT3, ERK, and MAPK signaling cascades [13,93,94]. Kuzu et al. recently reported that leelamine can interrupt important signaling pathways through the phosphorylation of several cell signaling proteins in melanoma cells [13]. There was an alteration in the RTK/AKT signaling pathway and the most important pathways, the PI3K/AKT and AKT/mTOR pathways, were modified after leelamine treatment (Figure 5). Indeed, the hypoactivation of RTKs followed by the effect of extracellular factors inactivates the PI3K/AKT, STAT, and MAPK signaling cascades. Additionally, the reduction of mutant V600E BRAF protein can inactivate the MAPK cascade without RTK effects [95,96]. Mutant Ras and BRAF genes have been found in some cancers, such as melanomas, and play a key role in the growth and spread of cancer cells [97]. STAT3 activity is significantly reduced by leelamine and subsequently, Bcl-2 and Bcl-xL expression levels decrease [13]. Leelamine is a lysosomotropic diterpene that causes autophagic flux disruption and the inhibition of signaling pathways in malignant cancers.

### 4.2. Stilbenoids

**Figure 6:**
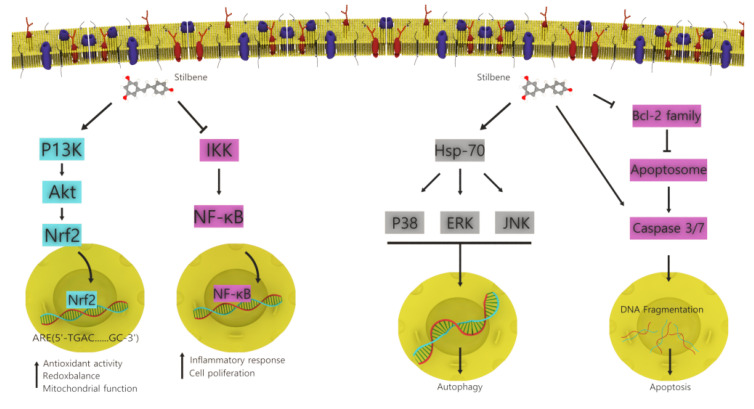
Mechanism of action of stilbenoids. Stilbenoids induces apoptosis through the caspase activation pathway; they also suppress Bcl-2 expression and apoptosome production. Stilbenoids induce autophagy in cancer cells in an hsp-70 dependent mechanism. These compounds suppress the NF-κB signaling pathway that plays an important role in inflammation. They also activate the Nrf2 antioxidant defense system (adopted with modification from [107]).

Stilbenoids are formed by the heterogeneous oligomerization of stilbene monomers, such as resveratrol, oxyresveratrol, and isorhapontigenin, and sometimes their glucosides with a molecular backbone, consisting of 1,2-diphenylethylene units [98,99]. Stilbenoids are extracted from various plants, such as those of the families of Welwitschiaceae and Gnetaceae. The compounds have unique structures, and stilbene glucosides and stilbene oligomers are found in *Gnetum latifolium* and *Welwitschia mirabilis*. Stilbenoids have several biological activities, such as anticancer, antibacterial, anti-inflammatory, antioxidant, and antiviral activities [100,101]. Previous studies have reported the growth inhibition activity of stilbenoids against human cancer cell lines. In human leukemia HL60 cells, DNA fragmentation and nuclear condensation have been observed. Among the 56 stilbenoids, gnemonol G and gnetin I demonstrated high anticancer activity that was approximately two-fold higher than that of resveratrol [34]. Among stilbenoids, resveratrol is the most well-known and commercially available product. It has demonstrated potent antioxidant, anticancer, anti-inflammatory, and atherosclerosis prevention properties [102]. However, other stilbenoids, such as viniferin, have stronger anticancer activity than resveratrol [103,104]. Figure 6 shows the mechanism of action of stilbenoids; the NF-κB signaling pathway plays a vital role in inflammation; resveratrol has been studied inhibiting this signaling pathway in the treatment of several diseases [105]. Resveratrol also has antioxidant properties through activation of the PI3K/Akt/Nrf2 intracellular signaling pathway and restored SOD and GSH levels [106]. Apoptosis induction by resveratrol has been associated with caspase induction and downregulation of Bcl-2. Recent studies have shown that stilbenoids induced tumor autophagy is an hsp70-dependent mechanism by lysosomal membrane permeabilization [107].

### 4.3. Pinenes

Pinenes (α- and β-enantiomers) are organic compounds derived from monoterpenes with the molecular formula C_10_H_16_. They are the most abundant terpenoids in nature and are mainly found in the essential oils of *Pinus* species, as well as in various other plants. α- and β-pinenes are extracted from different parts of pines, such as from the needles, nuts, pollen, and bark oils [20,108]. Pinenes have anti-inflammatory and antibacterial activities [109,110]. Both α- and β-pinenes have anticancer activity, and can inhibit the proliferation of human cancer cell lines, including breast, prostate, liver, and colon cancer cell lines [14,111]. Pinenes have good anticancer activity, arrest the cell cycle in the G2/M phase, and inhibit hepatoma cell proliferation [14]. *Pinus koraiensis* essential oils, mainly consisting of α-pinene, stop the proliferation and migration of human gastric carcinoma cells (MGC-803) and induce apoptosis [89]. This is due to its effect on the Hippo signaling pathway, known as the tumor suppressor, which has been extensively studied [112]. In this pathway, the phosphorylation of YAP occurs and inhibits the migration and proliferation of tumor cells [113]. α-Pinene can decrease the expression of YAP, leading to the inhibition of cancer cells and the induction of apoptosis [89].

### 4.4. Caryophyllenes

Sesquiterpenes have multiple biological activities, including anticancer, antibacterial, anti-inflammatory, and antiviral activities [20,114]. α-Caryophyllene, β-caryophyllene, and β-caryophyllene oxide are sesquiterpenes from terpenoids naturally isolated from the essential oils of pine species and many other aromatic plants. Caryophyllenes inhibit the proliferation of different types of cancer cells, such as those of liver, breast, lung, and prostate adenocarcinoma [90,115,116]. β-Caryophyllene suppresses STAT3 activation in human breast and prostate carcinoma and in multiple myeloma cell lines via the inactivation of IL-6 [36]. Src, JAK1, and JAK2 may be involved in β-caryophyllene-induced STAT3 inactivation. β-caryophyllene suppresses the PI3K/AKT signaling pathway, and therefore acts as a strong anticancer agent [90].

## 5. Conclusions and Future Development

For centuries, plants have been used as medicines for the treatment of human diseases in most regions worldwide. Recently, partly due to renewed public interest, scientific research efforts and medical communities around the world have produced considerable information regarding the pharmacological use, effects, and future of herbal medicine development and therapeutic phytochemicals for cancer treatment [117]. While considerable efforts have been made to promote and confirm the efficacy of many traditional therapies or various herbal formulations, well-defined clinical trials and systematic standardized research are still quite limited and should be carried out more extensively to accelerate the development of new phytomedicines [118]. In recent years, the FDA has approved botanical drugs, such as Veregen^®^ (a fraction of the green tea leaf water extract), for the treatment of patients with external genital warts and Crofelemer^®^ (a proanthocyanidin from the *Croton lechleri* tree latex) for diarrhea treatment in patients with HIV/AIDS [119,120]. This review summarizes the potential role of gymnosperms in the development of anticancer compounds (Table 2). Some potentially pharmacological compounds derived from gymnosperms could serve as new botanical drugs for cancer treatment.

## Figures and Tables

**Table 1:** Gymnosperm-derived commercially available products with therapeutic effects.

Commercially Available Product ^a^	Parental Plants	Common Name	Biological Activity	Mechanism of Anticancer Activity	References
Paclitaxel	*Taxus brevifolia*	Pacific yew	Anticancer effects	Microtubule inhibitor, induces apoptosis, Bcl-2 inhibitor	[12,23]
Pinostrobin	*Pinus strobus*	Eastern white pine	Anti-oxidative, anticancer, anti-inflammatory, and antimicrobial effects	Induces apoptosis, ROS generation in cancer cells, DNA fragmentation	[24,25,26,27]
Pinocembrin	*Pinus pinaster*	French maritime pine	Anticancer, antimicrobial, anti-inflammatory, and antioxidant effects	Increases the activity of heme oxygenase, caspase-3 and 9, and Bax	[28]
Pycnogenol	*Pinus pinaster*	French maritime pine	Anticancer, anti-inflammatory, antioxidant, blood clotting reduction, and LDL cholesterol-lowering effects	Increases nitric oxide levels in serum, inhibits NF-kB activity	[29,30,31,32]
Enzogenol	*Pinus radiata*	Monterey pine	Anticancer, anti-inflammatory, antioxidant, cardioprotective, and neuroprotective effects	Induces apoptosis, increases the activity of caspase-3, Bcl-2 inhibitor	[33]

^a^ Chemical structures of the commercially available products are shown in Figure 1.

**Table 2:** Gymnosperm-derived chemical compounds with therapeutic effects.

Chemical Compound *	Parental Plants	Biological Activity	Mechanism of Anticancer Activity	References
Leelamine	*Pinus* species	Anticancer	Disruption of cholesterol homeostasis and autophagic flux inhibitor	[13,87]
Stilbenoids	*Welwitschia mirabilis*	Anticancer, antibacterial, anti-inflammatory, and antioxidant	Apoptosis and growth inhibition	[34]
Pinenes	*Pinus* species	Anticancer, antibacterial, and anti-fungal	Cell-cycle arrest in the G2/M phase and induction of apoptosis	[14,88,89]
Caryophyllenes	*Pinus* species	Anticancer, antibacterial, and anti-inflammatory	Suppression of STAT3 activation and suppression of the PI3K/AKT signaling pathway	[36,90]

* All of chemicals are terpenoids and phenolic compounds.

## Data Availability

Not applicable.

## Abbreviations

Akt	protein kinase B
ASK1	apoptosis signal-regulating kinase 1
ATF6	activating transcription factor 6
Bak	B-cell lymphoma-2 homologous antagonist/killer
Bax	Bcl-2 associated X protein
Bcl-2	B cell lymphoma 2
Bcl-xL	B-cell lymphoma-extra-large
BIM	B-cell lymphoma-2-like protein 11
Cas	cellular apoptosis susceptibility protein
caspase	cysteine-aspartic proteases
CDKs	Cyclin-dependent kinases
CHOP	C/EBP-homologous protein
Cyt c	cytochrome c
ER	endoplasmic reticulum
ERK	extracellular signal-regulated kinase
FADD	Fas-associated protein death domain
G1	Gap 1 phase for cell enlargement
G2	Gap 2 phase
Hsp 70	heat shock protein 70
ICAM-1	intercellular adhesion molecule 1
IKK	NF-κB kinase
IRAK	interleukin-1 receptor associated kinase
IRE1	inositol-requiring enzyme 1
JNK	c-Jun-N terminal kinase
KSR	kinase suppressor of Ras
MAPK	mitogen-activated protein kinase
MEK	mitogen-activated protein kinase kinase
M phase	mitosis phase
mTOR	mammalian target of rapamycin
MYD88	myeloid differentiation primary response 88
NFκB	Nuclear factor-κB pathway
Nrf2	Nuclear factor erythroid 2-related factor 2
PI3K	phosphoinositide 3-kinase
PIP2	phosphatidylinositol-4, 5-bisphosphate
PIP3	phosphatidylinositol-3, 4, 5-triphosphate
Raf-1	Raf kinase family
ROS	reactive oxygen species
RTK	receptor tyrosine kinase
S phase	synthesis phase
STAT3	signal transducer and activator of transcription 3
TAT3	tyrosine aminotransferase 3
tBid	truncated Bid
TLR4	Toll-like receptor 4
TRAF	TNF receptor associated factor
TRIF	TIR-domain-containing adapter-inducing interferon-β
TRAM	TRIF-related adapter molecule
ULK1	unc-51-like kinase 1
VCAM-1	vascular cell adhesion molecule 1
Xbp1	X-box binding protein 1
